# Development of a Pressurized Green Liquid Extraction Procedure to Recover Antioxidant Bioactive Compounds from Strawberry Tree Fruit (*Arbutus unedo* L.)

**DOI:** 10.3390/plants12102006

**Published:** 2023-05-17

**Authors:** Anica Bebek Markovinović, Sanja Milošević, Nemanja Teslić, Branimir Pavlić, Predrag Putnik, Irena Brčić Karačonji, Karlo Jurica, Dario Lasić, Danijela Bursać Kovačević

**Affiliations:** 1 Faculty of Food Technology and of Biotechnology, University Zagreb, Pierottijeva 6, 10000 Zagreb, Croatia; abebekmarkovinovic@pbf.hr; 2 Faculty of Technology, University of Novi Sad, Blvd. Cara Lazara 1, 21000 Novi Sad, Serbia; sanjamilosevic9898@gmail.com (S.M.); bpavlic@uns.ac.rs (B.P.); 3 Institute of Food Technology, University of Novi Sad, Blvd. Cara Lazara 1, 21000 Novi Sad, Serbia; nemanja.teslic@fins.uns.ac.rs; 4 Department of Food Technology, University North, Trg dr. Žarka Dolinara 1, 48000 Koprivnica, Croatia; 5 Institute for Medical Research and Occupational Health, Ksaverska Cesta 2, 10000 Zagreb, Croatia; ibrcic@imi.hr; 6 Faculty of Health Studies, University of Rijeka, Viktora Cara Emina 5, 51000 Rijeka, Croatia; 7 Special Security Operations Directorate, Ministry of the Interior, Ulica Grada Vukovara 33, 10000 Zagreb, Croatia; juricakarlo@gmail.com; 8 Andrija Štampar Teaching Institute for Public Health, Mirogojska 16, 10000 Zagreb, Croatia; dario.lasic@stampar.hr

**Keywords:** strawberry tree fruit, phenolic compounds, antioxidant capacity, pressurized liquid extraction (PLE), green extraction, optimization

## Abstract

Strawberry tree fruits (*Arbutus unedo* L.) are a natural source of valuable biologically active compounds. Therefore, the aim of this work was to develop a green extraction approach using pressurized liquid extraction (PLE) to provide the highest yield of bioactive compounds. Different extraction solvents (distilled water, 50% and 96% ethanol) and different PLE parameters were investigated: temperature (40, 80, and 120 °C), static extraction time (5 and 10 min), and number of cycles (1 and 2). Total phenolic contents (TPC), hydroxycinnamic acids (HCA), flavonols (FL), total flavonoids (TF), condensed tannins (CT), and antioxidant capacity (DPPH and FRAP) were determined in PLE extracts. Solvent type, temperature, static extraction time, and number of cycles had a statistically significant effect on all bioactive compounds and antioxidant capacity (*p* ≤ 0.05). All bioactive compounds were positively correlated with temperature, except for TPC and DPPH. For all polyphenols studied, the optimal PLE parameters were a temperature of 120 °C, a static extraction time of 10 min and 2 cycles. The best solvent for most bioactive compounds was 96% ethanol, except for TPC, for which 50% ethanol was better. This study suggests that PLE is a promising tool to intensify the extraction of bioactive compounds from strawberry tree fruits.

## 1. Introduction

The strawberry tree (*Arbutus unedo* L.) is an evergreen Mediterranean shrub known for its health-promoting properties [1]. The fruits of the strawberry tree ripen several times in autumn and form a round fruit whose color ranges from green-yellow to orange-red depending on the stage of ripening [2,3].

Due to the high content of bioactive compounds, the fruits of the strawberry tree are characterized by strong antioxidant, anti-diabetic, antiproliferative, and antimicrobial properties [4]. In addition to the exceptional nutritional composition rich in sugars, unsaturated fatty acids, vitamins, proteins, organic acids, and dietary fibers, these fruits also have a high content of biologically active phenolic compounds, such as phenolic acids, flavonoids, anthocyanins, and condensed tannins [1,5]. In addition to phenolics, its health-promoting properties are also attributed to minerals. Studies have shown that strawberry tree fruits are a good source of calcium, magnesium, potassium, sodium, and phosphorus and low in potentially toxic elements (As, Cd, Cr, Hg, and Ni) [6].

Recently, consumers have become aware that a healthy lifestyle includes the consumption of functional foods, which have a positive effect on consumer health when consumed in appropriate amounts [7]. Strawberry tree fruits, a good bioresource with valuable chemical composition, are an excellent raw material for the development of functional foods. However, it is underutilized for these purposes because it tends to grow as a wild plant and is not cultivated [1].

Since the seasonal ripening of strawberry tree fruit limits its use, its high value ingredients could be extracted and further used for the development of functional foods. Currently, various extraction methods are increasingly used in the development of functional foods to isolate and obtain all the high-quality bioactive compounds [8]. Such extracts could be of particular value as natural food additives. Natural additives are of great value as an alternative to synthetic additives in the food industry because of their ability to color, preserve, and also enhance the bioactive properties of foods, without the hazardous effects associated with chemical synthetic additives [9]. For example, strawberry tree fruit extract, obtained by heat-assisted extraction (90 °C/5 min), which is rich in anthocyanins, was incorporated as a natural colorant in wafers, that exhibited various bioactive properties [10]. The addition of strawberry tree fruit extract to the limpets pâté prevented changes in color, texture, and pH value during 90 days of cold storage [11].

However, sustainable and environmentally friendly alternatives to traditional extraction methods, such as pressurized liquid extraction (PLE), have not yet been used for the extraction of bioactive compounds from strawberry tree fruits. Conventional extraction methods are expensive, less selective or have poor extraction yields, usually require longer extraction times, and often require the use of various toxic organic solvents such as methanol, acetone, etc. [12,13]. In addition, these techniques require subsequent removal of the toxic solvent from the extract. Recently, there has been much interest in developing new sustainable “green” extraction methods that use green solvents, such as water and/or ethanol [14,15]. PLE was shown as a suitable green approach to obtain multicomponent extracts consisting of different bioactive molecules that could exert synergistic protection against oxidation [16]. The application of this technology with water as the extraction solvent is particularly promising in pressurized hot water extraction (PHWE), where water remains in a liquid state when the temperature under the critical pressure rises above 100 °C. The dielectric constant (ε) of water then decreases significantly, making the properties of water similar to those of organic solvents and thus allowing compounds with low polarity to be extracted [14]. This category of sustainable technologies includes the PLE that uses a pressure between 5 and 15 MPa and a temperature between 50 and 200 °C to maintain the solvent in liquid states for short time periods (5–10 min). By using these pressure and temperature conditions, there is a change in the physicochemical properties of the solvent. For example, mass transfer rates are improved since the surface tension and viscosity of the solvent are reduced, whereas the solubility of the analytes is increased. This allows the solvent to penetrate more easily and deeply into the treated solid matrix. As a result, significantly higher extraction yields are achieved compared to conventional extractions. Therefore, PLE leads not only to faster extraction processes but also to lower solvent consumption during sample preparation of solids. In addition, most of the instruments used for PLE are automated, which allows the development of less labor-intensive methods and improves reproducibility [17].

Considering that the fruits of the strawberry tree are rich in various bioactive compounds with different chemical structures that also affect the antioxidant capacity, it is important to develop and optimize the extraction parameters that provide the best yield of these antioxidant bioactive compounds. Since the PLE technique has not yet been tested for the recovery of extracts from strawberry tree fruits, the aim of this work was to develop an environmentally friendly extraction technology that involves the use of generally recognized as safe (GRAS) solvents. To achieve this, research investigated different operating conditions of the PLE process such as: (i) the type of solvent, (ii) the temperature, (iii) the static extraction time, and (iv) the number of cycles on the recovery of antioxidant bioactive compounds from strawberry tree fruits. In addition, the extraction parameters of PLE will be optimized to obtain the maximum yield of the studied antioxidant bioactive compounds to identify optimal strawberry tree fruit extracts for the production of functional foods.

## 2. Results and Discussion

### 2.1. The Influence of PLE Extraction Parameters on Polyphenolic Compound Content and Antioxidant Potential

Table 1 shows the statistically processed data of the total phenolic content (TPC), hydroxycinnamic acids (HCA), flavonols (FL), total flavonoids (TF), condensed tannins (CT), and antioxidant capacities (DPPH and FRAP) depending on the PLE variables. The extraction variables were: solvent type (distilled water, 50% ethanol, and 96% ethanol); temperature (40 °C, 80 °C, and 120 °C); static extraction time (5 and 10 min); and number of cycles (1 and 2). The average value for TPC was 183.26 ± 1.44 mg 100 g^−1^. Izcara et al. [18] found TPC in berries of A. unedo L. to be 99.00 mg 100 g^−1^, which is a lower amount compared to our results, but the authors used a conventional solid–liquid extraction procedure with stirring. The highest representation in the data set was CT with an average of 44.23 ± 0.40 mg 100 g^−1^, followed by the HCA with 24.81 ± 0.24 mg 100 g^−1^, and FL and TF with 12.06 ± 0.18 mg 100 g^−1^ and 10.80 ± 0.05 mg 100 g^−1^, respectively. Pallauf et al. [19] obtained 1.14 ± 0.346 mg 100 g^−1^ flavonols and 27.46 ± 0.989 mg 100 g^−1^ proanthocyanidins by conventional extraction method using methanol as an extraction solvent. These results are drastically lower than those of the same category of compounds in our results, indicating the efficiency of the *PLE* method in terms of FL and CT recovery. However, it is very difficult to compare the results with other scientific results because PLE extraction efficacy has not yet been studied for A. unedo L. fruits.

The impact of PLE variables on the targeted responses was consistent, as all polyphenols followed exactly the same trend for all PLE parameters. For TPC, HCA, FL, TF, and CT the most convenient solvent was 96% ethanol and the most unsuitable was distilled water. Ethanol (96%), in comparison to other extraction solvents, yielded 4-times more TPCs, 11-times more HCA, 9.6-times more FL, 3-times more TF, and 4-times more CT. In addition to the other PLE parameters tested, the solvent polarity is a significant parameter affecting the efficiency of PLE extraction. Binary ethanol–water solvents were also previously found to affect the phenolic profile and antioxidant capacity in plant extracts [20]. Similar results were obtained by Scarano et al. [21], where a mixture of ethanol and water (7:3, *v*/*v*) gave the best yield of TPC from strawberry tree fruit compared to water. In addition, Jacotet-Navarro et al. [22] investigated the influence of ethanol to water ratio on the yield in the extraction of antioxidants from rosemary by reflux extraction. The authors found that increasing the ethanol concentration (from 0 to 60%) resulted in increased extraction yield (from 19 to 28%). Nonetheless, increasing the ethanol proportion from 60 to 80% had no significant effect. Beyond 80%, however, increasing the ethanol percentage was associated with a decrease in extraction yield (from 26 to 15%). Increasing the ethanol concentration (from 60 to 90%) in binary extraction solvents may alter the contribution of certain phenols to total phenols in plant extracts. Therefore, different data can be found in the literature depending on the plant source, as the profile of bioactive compounds plays an important role [20].

When considering the influence of temperature, it can be seen that for all tested polyphenol subclasses, higher temperature positively influences the higher extraction yield of all polyphenols in PLE extracts. In this research, 120 °C was the best choice for temperature, as opposed to 40 °C, which yielded the lowest concentration of the studied bioactive compounds. At this highest temperature setting, the yield of TPC was 1.3-fold higher, of HCA 2.2-fold higher, of FL 2.5-fold higher, of TF 1.6-fold higher, and of CT 1.9-fold higher. These results could be explained by the fact that higher temperatures lead to cell tissue rupture, which in turn results in acceleration and an increase in mass yield. In addition, an increase in temperature causes an acceleration of reaction kinetics, resulting in faster and enhanced recovery of the desired compounds [23]. Similarly, the longest static time resulted in the highest levels of all polyphenols, i.e., 10 min of static time yielded 1.1 times TPC, 1.2 times HCA, 1.3 times FL, 1.2 times TF, and 1.2 times CT. These results agree with the studies of Putnik et al. [24,25], who reported that increasing the static time of extraction resulted in improved recovery of TPC, TF, HCA, and FL from grape pomace skin and stevia leaves. Considering the number of cycles, ‘more was better’ as two cycles produced more of the compounds studied. Here, two cycles yielded 1.2 times more TPC, HCA and TF, and 1.1 times more FL and CT than extraction with 1 cycle. Each new cycle of PLE extraction, uses a fresh portion of the extraction solvent [17], which in the case of strawberry tree fruits has a positive effect on the better recovery of all bioactive compounds.

In summary and judging by the effect size (measured by the partial η^2^), the most important PLE variable for the extraction of all polyphenols from the strawberry tree fruit samples was the solvent type (96% ethanol), then the temperature (120 °C), the static time (10 min), and finally the number of cycles (2 cycles).

Strawberry tree fruit extracts have been shown to be an interesting source of secondary plant metabolites with antioxidant activity. In this study, average results for DPPH and FRAP assays of 223.61 ± 0.04 mg 100 g^−1^ and 994.03 ± 6.56 mg 100 g^−1^, respectively, were obtained for the PLE extracts (Table 1). The antioxidant assays gave similar results but with a lower degree of uniformity. Although 96% ethanol was the best solvent in terms of the highest antioxidant activity for both assays, distilled water outperformed 50% ethanol, which was the poorer solvent in this regard. Moreover, the differences between water and 96% ethanol were much less pronounced than for polyphenols. Longer extraction static times and an increase in temperature increased FRAP (as well as the other polyphenols) and decreased DPPH; a higher number of cycles increased FRAP and had no effect on DPPH (Table 1). This discrepancy in the results for antioxidant capacity could be due to different mechanisms by which bioactive compounds from the PLE extracts enter the reactions, which is a consequence of structure-activity relationships [26]. Because PLE was able to produce multicomponent extracts composed of bioactive molecules with different polarity, a different trend in the results for the determination of antioxidant capacity by two different methods that differ in the mechanisms of action is possible.

### 2.2. Correlation of Temperature with Polyphenolic Compounds and Antioxidant Capacity

In addition to the choice of suitable extraction conditions, temperature is one of the most important factors affecting the yield of the PLE extraction process because the physicochemical properties of the solvents are changed by increasing the temperature, for example, the polarity of the solvent can be adjusted by changing the temperature [17]. Temperature affects mass transfer properties by changing the surface tension of the solvent, diffusivity, and viscosity. Solubility of the analyte increases with the higher temperature, but can also improve the solubility of other compounds, transforming PLE into a less selective extraction. However, considering the thermolabile nature of polyphenolic compounds, high temperature can also have the opposite effect, i.e., it can cause degradation of these compounds. Therefore, it is important to carefully evaluate the manner in which temperature correlates with each group of polyphenolic compounds. Table 2 shows the correlation between polyphenolic compounds, antioxidant capacities, and temperature.

Temperature correlated with almost all polyphenolic subgroups and antioxidant capacities except TPC. The temperature did not correlate with TPC, whereas it correlated positively with all other polyphenolic compounds; more specifically, the increase in temperature contributed to the increase in the content of HCA, FL, TF, and CT. A similar trend was observed by Bursać Kovačević et al. [14], where the increase in temperature from 100 °C to 160 °C correlated positively with the content of CT and TPC. As with most other polyphenolic compounds (except TPC), temperature correlated positively with FRAP. Surprisingly, temperature setting had an opposite trend for DPPH; more precisely, as temperature increased, DPPH levels decreased (negative correlation). Moreover, FRAP followed the trend of the other polyphenols (TPC, HCA, FL, TF, and CT), with DPPH in an inversely proportional relationship to the polyphenolic compounds. In other words, with increasing levels of polyphenols, FRAP increased while DPPH decreased (Table 2). Numerous scientific studies have demonstrated the correlation between high bioactive compound content and high antioxidant activity [27,28], suggesting that changes in the proportion of bioactive compounds will be reflected in changes in the antioxidant capacity of the samples. Yang et al. [29] showed that the choice of solvent significantly affected the correlation of antioxidant capacities with TPC values, which may possibly be the reason for the disproportion of correlations between DPPH and FRAP and bioactive compounds. Moreover, Thaipong et al. [30], comparing ABTS, DPPH, FRAP and ORAC methods for antioxidant capacity analysis, concluded that it is necessary to perform at least two of the mentioned assays to ensure a higher level of accuracy of the results and to determine their correlation and follow-up. Considering the opposite tendencies of DPPH and FRAP methods in our results, it would be necessary to make additional efforts and tests in the future to make this tendency more precise.

### 2.3. Optimization of PLE Processing Parameters for Antioxidant Bioactive Compounds Extraction

Considering that strawberry tree fruits are a high-value source of bioactive compounds that can be added to other food products for their fortification, production of functional foods, nutraceuticals, cosmetic products, or other purposes, it is important to know the optimal extraction conditions under which the best yield of these compounds is obtained. Therefore, the parameters of PLE extraction were optimized to obtain the maximum yield of the mentioned bioactive compounds. The results are shown in Table 3.

For all polyphenols studied, the optimal PLE parameters were a temperature of 120 °C and a static extraction time of 10 min with 2 cycles. For TPC, the best solvent was 50% ethanol, whereas for the other bioactive compounds, the best solvent was 96% ethanol. This was in complete agreement with the MANOVA data.

**Figure 1:**
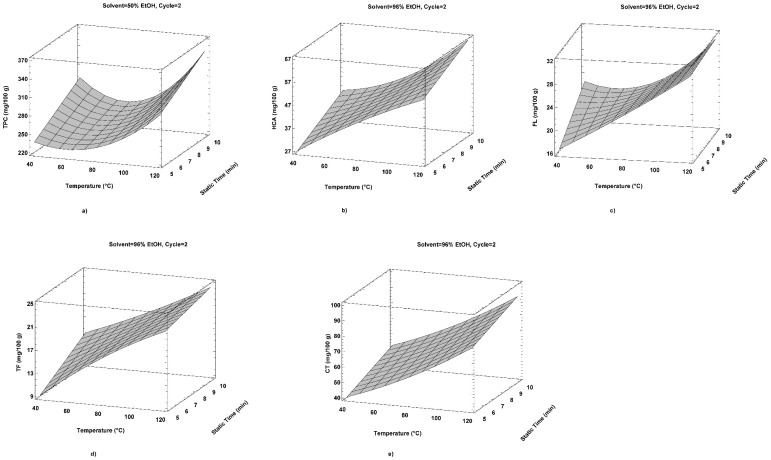
The impact of PLE operating conditions on examined polyphenols: (**a**) TPC-total phenolic content (mg 100 g^−1^); (**b**) HCA-hydroxycinnamic acids (mg 100 g^−1^); (**c**) FL-flavonols (mg 100 g^−1^); (**d**) TF-total flavonoids (mg 100 g^−1^); and (**e**) CT-condensed tannins (mg 100 g^−1^).

Figure 1 shows a graphic representation of the optimal PLE extraction parameters for each subgroup of bioactive compounds studied.

The choice of a suitable solvent is one of the most important parameters for successful extraction. Of the three solvents tested, water proved to be the most polar solvent, and 96% ethanol the least polar solvent, while 50% ethanol the most suitable for maximum yield of TPC. These results are consistent with those of Pollini et al. [31], who showed that 50% ethanol gave a better yield of TPC from fresh apple pomace than 30% and 70% ethanol at 40 °C. Yang et al. [29] stated that strongly polar water and relatively weakly polar acetone do not facilitate the extraction of TPC from mulberry extract, but appropriate polar solvents do. Additionally, the authors noted that the extraction depends on the comprehensive characteristics of the solvent and that the addition of acid to the extraction solvents positively affects the yield of TPC, which may serve as a guide for further studies. All other bioactive compounds (HCA, FL, TF, and CT) achieved the maximum extraction yield with 96% ethanol as solvent (Table 3).

As for the other parameters of PLE extraction (temperature, static extraction time, and number of cycles), the highest levels of bioactive compounds were obtained at the maximum experimental values (120 °C, 10 min, and 2 cycles). Herrero et al. [32] performed the optimization of PLE parameters of antioxidants from the microalga *Spiruline platensis* and obtained results following our trend. The maximum extraction yield was obtained at the highest experimental temperature of 170 °C and the highest extraction time of 15 min. In addition, Bursać Kovačević et al. [14] examined the TPC yield of pressurized hot water extraction parameters (temperature 100–160 °C; static extraction time 5–10 min and number of cycles 1–3) from *Stevia rebaudiana* Bertoni leaves and obtained the results that three cycles of 5 min and two cycles of 10 min static time provide the best yield. Accordingly, a shorter time at higher temperatures and a longer time at lower temperatures leads to a better yield of phenolic compounds, suggesting the connection and optimization of these parameters [33]. Considering that our results indicate the highest yield of bioactive compounds at the maximum values of all PLE extraction parameters (temperature, static extraction time, and number of cycles), this suggests that the critical values of the given parameters were not exceeded, and, therefore, it was not necessary to study their connection and coordination. Additionally, the possibility of increasing the value of the PLE parameters remains here for further investigation and monitoring of their connection and coordination.

## 3. Materials and Methods

### 3.1. Chemical and Standards

HPLC 99% pure methanol was obtained from Honeywell (Paris, France). Folin-Ciocalteau (FC) reagent was purchased from Fisher Scientific UK (Loughborough, UK). Sodium carbonate, anhydrous (99.5–100.5%), hydrochloric acid (37%, *w*/*w*), sulfuric acid (96%, p.a.), and formic acid (98%, p.a.) were purchased from Lach-Ner (Neratovice, Czech Republic). Chlorogenic acid (min. 95%) and vanillin (99%) were purchased from Thermo Fisher (Kandel, Germany). Sodium acetate trihydrate resistant to potassium permanganate and iron (III)-chloride hexahydrate were purchased from Kemika (Zagreb, Croatia). Ethanol (96% pure) was obtained from Gram-mol (Zagreb, Croatia). Potassium acetate (99%), quercetin (95%), and aluminum chloride (98.5%) were obtained from Acros Organics (Guangzhou, China). DPPH (2,2-diphenyl-1-picrylhydrazyl radical), TPTZ (2,4,6-tris-2-pyridyl-*s*-triazine), and gallic acid standard (97.5–102.5%) were purchased from Sigma-Aldrich (St. Louis, MO, USA). Glacial acetic acid (≥99.8%) was obtained from Honeywell Fluka^TM^ (Seelze, Germany), and Trolox standard (6-hydroxy-2,5,7,8-tetramethylchroman-2-carboxylic acid) used for DPPH and FRAP method was obtained from Biosynth (Bratislava, Slovakia).

### 3.2. Plant Material

Strawberry tree fruits (*Arbutus unedo* L.) were harvested in October 2022, on the island of Mali Lošinj (44°32′ N 14°28′ E/44.53° N 14.46° E), Croatia, and brought to the laboratory. After washing with tap water, the fruits were drained with cellulose paper and frozen in polypropylene bags at −18 °C until PLE procedure.

#### Pressurized Liquid Extraction (PLE)

Prior to PLE, frozen strawberry tree fruits were thawed at room temperature, ground in a household blender, and homogenized. PLE was performed using an accelerated solvent extractor (ASE 350, Dionex, Sunnyvale, CA, USA) and 22 mL stainless steel extraction cells. A cellulose or glass fiber filter was placed on the bottom of each extraction cell. The cellulose filter was used for extractions with aqueous ethanol (50% and 96%), while the glass fiber filter was used for the PLE with water as the extraction solvent. In each experimental trial, 4 (±0.001) g of strawberry tree fruits were mixed with 1 g of diatomaceous earth in a glass beaker and placed in an extraction cell. Extractions were performed at fixed pressure (~10 MPa), solvent rinse volume (50%), and flushing time with nitrogen (90 s). On the other hand, temperature (40, 80, and 120 °C), static extraction time (5 and 10 min), number of extraction cycles (1 and 2), and solvent type (water, 50% and 96% aqueous ethanol; *v*/*v*) were independent PLE variables. A total of 36 different extraction setups were performed (Table 4). After extraction, all samples were diluted with fresh solvent to adjust solid/liquid ratio to 1:15 (*w/v*). Finally, the obtained extracts were collected in 50 mL conical tubes and stored at −20 °C before analysis.

### 3.3. Determination of Total Phenolic Content (TPC)

Total phenolic content was measured using the Folin–Ciocalteu modified spectrophotometric method known from the literature [34]. Briefly, 400 mL of the previously appropriately diluted extract was mixed with 400 µL of the FC reagent (previously diluted 5-fold with distilled water) and 4 mL of a 7.5% sodium carbonate solution (*w/v*). The reaction mixture was allowed to stand at room temperature for 20 min and then the absorbance of the reaction was measured at 725 nm using an LLG-uniSPEC 2 Spectrophotometer (Lab Logistics Group GmbH, Meckenheim, Germany). Duplicate measurements were performed for each sample. A calibration curve generated with different concentrations of gallic acid solutions (10–250 mg L^−1^) was used to calculate TPC, and the results were expressed as mg gallic acid equivalent (GAE) per 100 g of sample.

### 3.4. Determination of Total Flavonoids Content (TF)

For the determination of TF, a modified spectrophotometric method described in the literature was used [35]. Briefly, 0.5 mL of the extract, 1.5 mL of 96% ethanol, 0.1 mL of 10% aluminum chloride, 0.1 mL of 1 M potassium acetate, and 2.8 mL of distilled water were mixed, respectively. The blank sample is prepared in the same way, but instead of the extract, an extraction solvent was used, and instead of 10% aluminum chloride, distilled water was added. The reaction mixture was homogenized for 1 min on a vortex shaker (Grant Instruments Ltd., Cambs, UK), and the reaction mixture was allowed to stand at room temperature for 30 min. After that, the absorbance of the reaction was measured at 415 nm using an LLG-uniSPEC 2 Spectrophotometer (Lab Logistics Group GmbH, Meckenheim, Germany). Duplicate measurements were performed for each sample. A calibration curve prepared with different concentrations of quercetin standard solution (10–200 mgL^−1^) was used to calculate TF, and results were expressed as mg quercetin equivalent (QE) per 100 g of sample.

### 3.5. Determination of Total Hydroxycinnamic Acids (HCA) and Total Flavonols Content (FL)

HCA and FL were determined using a modified spectrophotometric method described in the literature [36]. A volume of 250 µL of the extract was mixed with 250 µL of solution 1 (1 g L^−1^ HCl solution dissolved in 96% ethanol) and 4.55 mL of solution 2 (2 g L^−1^ HCl dissolved in distilled water). The reaction mixture was homogenized on a vortex shaker (Grant Instruments Ltd., Cambs, UK) for 1 min and allowed to stand in the dark at room temperature for 30 min. Subsequently, the color response at 320 nm was measured for the determination of HCA and the color response at 360 nm was measured for the determination of FL using an LLG-uniSPEC 2 Spectrophotometer (Lab Logistics Group GmbH, Meckenheim, Germany). The blank sample was prepared in the same way, but an extraction solvent was used instead of the extract. Duplicate measurements were performed for each sample. HCA content was calculated using a calibration curve prepared with different concentrations of chlorogenic acid solution (10–600 mg L^−1^), and FL content was calculated with different concentrations of quercetin solution (10–600 mg L^−1^). The results of the HCA content and FL were expressed as mg chlorogenic acid equivalent (CAE) per 100 g of sample and as mg quercetin equivalent (QE) per 100 g of sample, respectively.

### 3.6. Determination of Condensed Tannins (CT)

A modified spectrophotometric method from the literature was used to determine CT [37]. A volume of 2.5 mL of reagent 1 (25% H_2_SO_4_ solution in methanol) was mixed with reagent 2 (1% vanillin solution in methanol) and 1 mL of extract. The reaction mixture was homogenized for 1 min using a vortex shaker (Grant Instruments Ltd., Cambs, UK) and then allowed to react for 10 min at room temperature. The absorbance of the reaction was then measured at 500 nm using an LLG-uniSPEC 2 Spectrophotometer (Lab Logistics Group GmbH, Meckenheim, Germany). The blank sample was prepared in the same way, but an extraction solvent was used instead of the extract. Duplicate measurements were performed for each sample. A calibration curve prepared with different concentrations of catechin standard solutions (10–120 mg L^−1^) was used to calculate CT, and the results were expressed as mg catechin equivalent (CA) per 100 g or 100 mL of the sample.

### 3.7. Determination of In Vitro Antioxidant Capacity

#### 3.7.1. 2,2-Diphenyl-1-picrylhydrazyl Method (DPPH)

Antioxidant activity was determined by the DPPH spectrophotometric method described in the literature [38]. Briefly, 1.5 mL of the extract and 3 mL of a 0.5 mM DPPH methanolic solution were mixed. The reaction mixture was allowed to react for 20 min at room temperature in the dark. As a control, 1.5 mL of 100% methanol and 3 mL of 0.5 mM DPPH solution were mixed. Methanol (100%) was used as a blank. Then, the absorbance of the color reaction was measured at 517 nm. Duplicate measurements were performed for each sample. A calibration curve generated with different concentrations of Trolox solution (10–150 µM) was used to calculate the antioxidant activity, and the results were expressed as mg Trolox equivalents (TE) per 100 g of the sample.

#### 3.7.2. Ferric Reducing Antioxidant Power Method (FRAP)

The second method used to determine antioxidant activity was the spectrophotometric FRAP method described in the literature [39]. The FRAP reagent was prepared by mixing 50 mL of acetate buffer (0.3 M) at pH 3.6, 5 mL of tripyridyltriazine (TPTZ) solution 10 mM prepared in HCl (40 mM) and 5 mL of ferric chloride solution (FeCl_3_) (20 mM). Briefly, 600 μL of the previously appropriately diluted extract and 4.5 mL of the FRAP reagent were added to the glass tubes. Then, the reaction mixture was homogenized on a vortex shaker (Grant Instruments Ltd., Cambs, UK) for 1 min and thermostatted in a water bath at 37 °C for 10 min. Then, the absorbance of the reaction was measured at 593 nm using an LLG-uniSPEC 2 Spectrophotometer (Lab Logistics Group GmbH, Meckenheim, Germany). The blank sample was prepared in the same way, but an extraction solvent was used instead of the extract. To calculate the antioxidant activity, a calibration curve was prepared with different concentrations of Trolox solution (10–150 µM) and the results were expressed as mg Trolox equivalents (TE) per 100 g of the sample.

### 3.8. Statistical Analysis

Experiments were designed as full factorial randomized experimental designs (n = 72) (Table 4). Dependent variables were the levels of: (i) total phenols (TPC; mg 100 g^−1^); (ii) hydroxycinnamic acids (HCA; mg 100 g^−1^); (iii) flavonols (FL; mg 100 g^−1^); (iv) total flavonoids (TF; mg 100 g^−1^); (v) condensed tannins (CT; mg 100 g^−1^); (vi) DPPH assay (mg 100 g^−1^); and (vii) FRAP assay (mg 100 g^−1^). Independent variables were: (i) cycle number (1 and 2); (ii) extraction temperature (40, 80, and 120 °C); (iii) duration of static extraction (5 and 10 min); and (iv) type of solvent (distilled water, 50% ethanol, and 96% ethanol). Descriptive statistics were used to evaluate the basic information on the experimental data set. Differences between treatments (continuous variables) were tested by multivariate analysis of variance (four-way ANOVA). The Pearson coefficient was used to assess correlations between pairs of continuous variables. Significance levels for rejection of a null hypothesis were α ≤ 0.05 for all tests. Analyses were performed using IBM SPSS Statistics (v.24), and experimental design was performed using Statgraphics Centurion^®^ (StatPoint Technologies, Inc., Warrenton, VA, USA).

## 4. Conclusions

The results show that pressurized liquid extraction (PLE) is an effective way to obtain “green” extracts rich in high-value bioactive compounds from strawberry tree fruits. The type of solvent, temperature, static extraction time, and number of cycles statistically significantly affected the yield of all bioactive compounds studied, as well as antioxidant capacity. An increase in temperature, static extraction time, and number of cycles was associated with an increase in the yield of bioactive compounds. Accordingly, the increase in temperature correlated statistically significantly with almost all the bioactive compounds studied (with the exception of total phenolic content), the increase in FRAP (as with the other polyphenols), and the decrease in DPPH.

By optimizing the extraction PLE process parameters, it was found that temperatures of 120 °C, a static extraction time of 10 min, and two cycles gave the highest yield of all bioactive compounds. The highest yield of bioactive compounds was obtained with 96% ethanol as an extraction solvent, except for total polyphenols, whose highest yield was recorded with 50% ethanol. Ultimately, it was demonstrated how liquid extraction under pressure can be used as a sustainable, environmentally friendly technology to obtain extracts rich in bioactive compounds from strawberry tree fruits. Due to their potent biological effects, strawberry tree fruits may provide a wide range of applications in the fortification of various foods, production of new functional foods, dietary supplements, cosmetics, and other purposes. Considering the obtained results, the PLE extraction method could be recommended as an environmentally safe method for the extraction of bioactive compounds with different polarities to obtain multicomponent extracts that could exert their synergistic effects.

## Figures and Tables

**Table 1:** Polyphenol content and antioxidant capacity in PLE strawberry tree fruit extracts.

PLE Variables	n	TPC ^1^	HCA ^2^	FL ^3^	TF ^4^	CT ^5^	DPPH ^6^	FRAP ^7^
Solvent Type		*p* ≤ 0.01 ^†^	*p* ≤ 0.01 ^†^	*p* ≤ 0.01 ^†^	*p* ≤ 0.01 ^†^	*p* ≤ 0.01 ^†^	*p* ≤ 0.01 ^†^	*p* ≤ 0.01 ^†^
Distilled water	24	58.95 ± 2.49 ^c^	4.05 ± 0.41 ^c^	2.47 ± 0.32 ^c^	5.86 ± 0.08 ^c^	14.83 ± 0.40 ^c^	224.07 ± 0.07 ^b^	798.01 ± 11.35 ^b^
50% EtOH	24	253.83 ± 2.49 ^a^	26.2 ± 0.41 ^b^	10.02 ± 0.32 ^b^	9.77 ± 0.08 ^b^	55.77 ± 0.40 ^b^	220.93 ± 0.07 ^c^	1221.1 ± 11.35 ^c^
96% EtOH	24	237.01 ± 2.49 ^b^	44.18 ± 0.41 ^a^	23.70 ± 0.32 ^a^	16.78 ± 0.08 ^a^	62.68 ± 0.40 ^a^	225.82 ± 0.07 ^a^	962.93 ± 11.35 ^a^
Temperature		*p* ≤ 0.01 ^†^	*p* ≤ 0.01 ^†^	*p* ≤ 0.01 ^†^	*p* ≤ 0.01 ^†^	*p* ≤ 0.01 ^†^	*p* ≤ 0.01 ^†^	*p* ≤ 0.01 ^†^
40 °C	24	161.24 ± 2.49 ^c^	16.25 ± 0.41 ^c^	7.43 ± 0.32 ^c^	7.75 ± 0.08 ^c^	31.27 ± 0.40 ^c^	226.23 ± 0.07 ^a^	774.28 ± 11.35 ^c^
80 °C	24	179.44 ± 2.49 ^b^	22.15 ± 0.41 ^b^	10.26 ± 0.32 ^b^	10.24 ± 0.08 ^b^	44.13 ± 0.40 ^b^	224.29 ± 0.07 ^b^	913.95 ± 11.35 ^b^
120 °C	24	209.11 ± 2.49 ^a^	36.03 ± 0.41 ^a^	18.50 ± 0.32 ^a^	14.41 ± 0.08 ^a^	57.88 ± 0.40 ^a^	220.31 ± 0.07 ^c^	1293.9 ± 11.35 ^a^
Static Extraction Time		*p* ≤ 0.01 ^†^	*p* ≤ 0.01 ^†^	*p* ≤ 0.01 ^†^	*p* ≤ 0.01 ^†^	*p* ≤ 0.01 ^†^	*p* ≤ 0.01 ^†^	*p* ≤ 0.01 ^†^
5 min	36	173.79 ± 2.04 ^b^	22.61 ± 0.34 ^b^	10.67 ± 0.26 ^b^	9.94 ± 0.06 ^b^	41.18 ± 0.33 ^b^	224.64 ± 0.05 ^a^	944.63 ± 9.27 ^b^
10 min	36	192.73 ± 2.04 ^a^	27.01 ± 0.34 ^a^	13.45 ± 0.26 ^a^	11.67 ± 0.06 ^a^	47.68 ± 0.33 ^a^	222.58 ± 0.05 ^b^	1043.4 ± 9.27 ^a^
Number of Cycles		*p* ≤ 0.01 ^†^	*p* ≤ 0.01 ^†^	*p* ≤ 0.01 ^†^	*p* ≤ 0.01 ^†^	*p* ≤ 0.01 ^†^	p = 0.23 ^‡^	*p* ≤ 0.01 ^†^
1	36	169.88 ± 2.04 ^b^	22.78 ± 0.34 ^b^	11.36 ± 0.26 ^b^	9.99 ± 0.06 ^b^	41.39 ± 0.33 ^b^	223.56 ± 0.05 ^a^	862.89 ± 9.27 ^b^
2	36	196.65 ± 2.04 ^a^	26.85 ± 0.34 ^a^	12.76 ± 0.26 ^a^	11.62 ± 0.06 ^a^	47.46 ± 0.33 ^a^	223.66 ± 0.05 ^a^	1125.2 ± 9.27 ^a^
Dataset average	72	183.26 ± 1.44	24.81 ± 0.24	12.06 ± 0.18	10.80 ± 0.05	44.23 ± 0.40	223.61 ± 0.04	994.03 ± 6.56

Results are expressed as mean ± standard error. Values represented with different letters are statistically different at *p* ≤ 0.05; ^†^ significant factor in multifactor analysis; ^‡^ not significant factor in multifactor analysis. ^1^ TPC—total phenolic content (mg 100 g^−1^); ^2^ HCA—hydroxycinnamic acids (mg 100 g^−1^); ^3^ FL—flavonols (mg 100 g^−1^); ^4^ TF—total flavonoids (mg 100 g^−1^); ^5^ CT—condensed tannins (mg 100 g^−1^); ^6^ DPPH assay (mg 100 g^−1^); and ^7^ FRAP assay (mg 100 g^−1^).

**Table 2:** Mutual correlations among polyphenols, antioxidant capacities, and temperature.

	T	TPC	HCA	FL	TF	CT	DPPH	FRAP
T	1.00	0.21	0.40 *	0.40 *	0.47 *	0.43 *	−0.58 *	0.62 *
TPC		1.00	0.80 *	0.67 *	0.71 *	0.92 *	−0.27 *	0.64 *
HCA			1.00	0.95 *	0.95 *	0.91 *	−0.19	0.48 *
FL				1.00	0.93 *	0.80 *	−0.18	0.35 *
TF					1.00	0.85 *	−0.12	0.46 *
CT						1.00	−0.28 *	0.66 *
DPPH							1.00	−0.57 *
FRAP								1.00

* Correlation is significant at the *p* ≤ 0.05. T—temperature; TPC—total phenolic content (mg 100 g^−1^); HCA—hydroxycinnamic acids (mg 100 g^−1^); FL—flavonols (mg 100 g^−1^); TF—total flavonoids (mg 100 g^−1^); CT—condensed tannins (mg 100 g^−1^); DPPH assay (mg 100 g^−1^); and FRAP assay (mg 100 g^−1^).

**Table 3:** Optimal PLE parameters for maximal extraction of antioxidant bioactive compounds.

PLE Variables	TPC ^1^	HCA ^2^	FL ^3^	TF ^4^	CT ^5^
Extraction Solvent	50% EtOH	96% EtOH	96% EtOH	96% EtOH	96% EtOH
Temperature (°C)	120	120	120	120	120
Static Extraction Time (min)	10	10	10	10	10
Number of Cycles	2	2	2	2	2
Predicted values (mg 100 g^−1^)	351.65	66.36	31.06	24.29	92.24

^1^ TPC-total phenolic content (mg 100 g^−1^); ^2^ HCA-hydroxycinnamic acids (mg 100 g^−1^); ^3^ FL-flavonols (mg 100 g^−1^); ^4^ TF-total flavonoids (mg 100 g^−1^); and ^5^ CT-condensed tannins (mg 100 g^−1^).

**Table 4:** Pressurized-liquid extraction parameters used to recover antioxidant bioactive compounds from strawberry tree fruits.

Run	Solvent Type	Temperature (°C)	Static Extraction Time (min)	Number of Cycles
1	Distilled water	40	5	1
2	Distilled water	40	5	2
3	Distilled water	40	10	1
4	Distilled water	40	10	2
5	Distilled water	80	5	1
6	Distilled water	80	5	2
7	Distilled water	80	10	1
8	Distilled water	80	10	2
9	Distilled water	120	5	1
10	Distilled water	120	5	2
11	Distilled water	120	10	1
12	Distilled water	120	10	2
13	50% EtOH ^1^	40	5	1
14	50% EtOH	40	5	2
15	50% EtOH	40	10	1
16	50% EtOH	40	10	2
17	50% EtOH	80	5	1
18	50% EtOH	80	5	2
19	50% EtOH	80	10	1
20	50% EtOH	80	10	2
21	50% EtOH	120	5	1
22	50% EtOH	120	5	2
23	50% EtOH	120	10	1
24	50% EtOH	120	10	2
25	96% EtOH ^2^	40	5	1
26	96% EtOH	40	5	2
27	96% EtOH	40	10	1
28	96% EtOH	40	10	2
29	96% EtOH	80	5	1
30	96% EtOH	80	5	2
31	96% EtOH	80	10	1
32	96% EtOH	80	10	2
33	96% EtOH	120	5	1
34	96% EtOH	120	5	2
35	96% EtOH	120	10	1
36	96% EtOH	120	10	2

^1^ 50% EtOH—50% ethanol; ^2^ 96% EtOH—96% ethanol.

## Data Availability

Not applicable.
